# Extraskeletal Chondroma in the Middle Ear: A Case Report

**DOI:** 10.7759/cureus.98702

**Published:** 2025-12-08

**Authors:** Hiroshi Hyakusoku, Toshihide Inagi, Aritomo Yamazaki, Fumiyuki Goto, Koichiro Wasano

**Affiliations:** 1 Otorhinolaryngology, Yokosuka Kyosai Hospital, Yokosuka, JPN; 2 Otolaryngology – Head and Neck Surgery, Tokai University School of Medicine, Isehara, JPN

**Keywords:** conductive hearing loss, extraskeletal chondroma, middle ear tumor, soft tissue chondroma, transcanal endoscopic ear surgery

## Abstract

Extraskeletal chondroma, also known as soft tissue chondroma, is a benign cartilaginous tumor most commonly found in the hands and feet. The occurrence of extraskeletal chondroma in the external auditory canal is extremely rare. We report the first case of extraskeletal chondroma arising in the left middle ear. A 68-year-old female was referred to our department with a diagnosis of a left external - middle ear tumor accompanied by left-sided hearing loss. Otoscopic examination revealed a mass contacting the posterior surface of the anterosuperior quadrant of the tympanic membrane, protruding outward into the external ear canal. Computed tomography imaging demonstrated a soft tissue mass occupying the attic and tympanic cavity, extending into the external ear canal, without evidence of ossicular or bony erosion. The tumor was removed surgically using transcanal endoscopic ear surgery under general anesthesia. In addition to the prominent mass observed from the external ear canal, several smaller white and translucent tumors were identified deeper within the tympanic cavity and were meticulously removed individually. Careful monitoring may be appropriate in asymptomatic cases because malignant transformation of extraskeletal chondroma is rare. While recurrence of chondromas in the external auditory canal has not been reported, recurrence and malignant transformation have been observed in other anatomical sites. Therefore, long-term follow-up is necessary to monitor for potential recurrence or pathological progression.

## Introduction

Extraskeletal chondroma, also known as soft tissue chondroma, is a benign cartilaginous tumor most commonly found in the hands or feet [1]. It typically occurs between the ages of 2 and 70 years old and shows a slight male predominance [2]. The occurrence of extraskeletal chondroma in the external auditory canal is extremely rare. Here, we report, to our knowledge, the first documented case of an extraskeletal chondroma arising in the left middle ear.

## Case presentation

**Figure 1:**
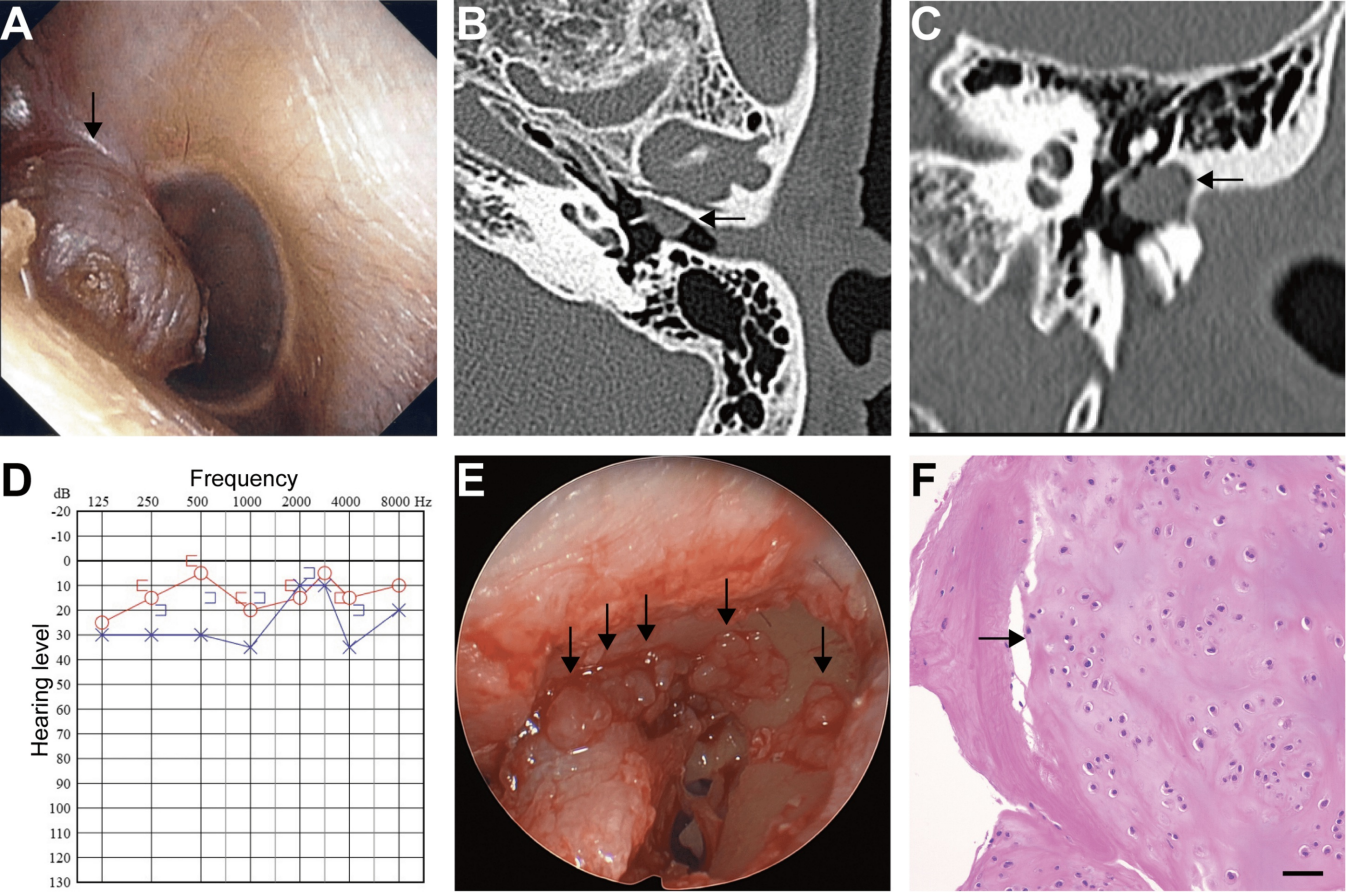
Images of the present case (A) Otoscopic view of the left external ear canal (arrow). Computed tomography revealed a space-occupying lesion in the anterosuperior quadrant of the tympanic membrane (B: axial, C: coronal, arrows); (D) Pre-operative audiogram; (E) The tumors in the tympanic cavity of surgical findings (arrows); (F) The portion of the biggest tumor is shown. The tumor was histologically formed from hyaline cartilage (arrow). Hematoxylin and eosin stain. Scale bar: 200 μm.

A 68-year-old female was referred to Yokosuka Kyosai Hospital with a diagnosis of a left external - middle ear tumor accompanied by left-sided hearing loss. Otoscopic examination revealed an elastic and soft mass contacting the posterior surface of the anterosuperior quadrant of the tympanic membrane, protruding outward into the external ear canal (Figure 1A). Computed tomography (CT) imaging demonstrated a soft tissue mass occupying the attic and tympanic cavity, extending into the external ear canal, without evidence of ossicular or bony erosion (Figures 1B, 1C). Pure-tone audiometry indicated conductive hearing loss on the left side (Figure 1D). Surgical excision of the middle ear tumor was planned using transcanal endoscopic ear surgery under general anesthesia. Following elevation of the tympanomeatal flap, the tumor was visualized within the tympanic cavity. In addition to the prominent mass observed in the external ear canal, several smaller, white, and translucent tumors were identified deeper within the tympanic cavity and were meticulously removed individually (Figure 1E). The tumor was attached to the malleus but not fused and could be easily separated. Because the tumor was adherent to the tympanic membrane, resection included a portion of the tympanic membrane and overlying skin of the external ear canal to ensure clear margins. The chorda tympani nerve and the ossicular chain were preserved. Tympanic membrane and canal wall reconstruction was performed using an underlay technique with subcutaneous tissue harvested from the retroauricular region. Histopathological examination confirmed the diagnosis of extraskeletal chondroma (Figure 1F). All tumors exhibited the same histological features. At the one-year follow-up, no evidence of tumor recurrence was observed.

## Discussion

Extraskeletal chondroma most commonly occurs in the hands and feet. According to Chung et al., approximately 2% of cases arise in the head and neck region [1]. Within the ear, some cases have been reported in the external auditory canal and auricle, with the external auditory canal being the more frequently affected site [2,3]. However, to our knowledge, no previous cases of extraskeletal chondroma arising in the middle ear have been reported.

Lee et al. speculated that extraskeletal chondromas may originate from heterotopic cartilaginous embryonic rests of Meckel’s cartilage, which is derived from the first branchial arch [4]. Although Meckel’s cartilage largely regresses during development, remnants contribute to the formation of the malleus and incus. The bones of the external auditory canal also develop through ossification of cartilage derived from the first branchial arch. These embryological origins may explain the occurrence of extraskeletal chondromas in the external auditory canal or in proximity to the ossicles. Furthermore, it has been reported that these tumors frequently arise in the anterior portion of the medial segment of the bony external auditory canal, particularly just anterior to the short process of the malleus [2]. The predilection for such specific anatomical sites lends support to the embryological hypothesis proposed by Lee et al.

In the present case, the tumor was identified in the anterior-superior quadrant of the tympanic membrane. It is plausible that, as the lesion enlarged, it extended further into the tympanic cavity. Notably, whereas previously reported cases have typically described solitary tumors, this case was characterized by the presence of multiple lesions. This atypical presentation may suggest a distinct pathogenesis or represent a previously unrecognized variation in the behavior of extraskeletal chondromas involving the middle ear.

Differential diagnoses for chondroma of the external auditory canal include keratoma, exostosis, osteoma, and ossifying fibroma, due to the typically bony-white appearance and hard consistency of such lesions [5,6]. However, in the present case, visual inspection did not reveal the characteristic hard, white tissue commonly seen in these entities. Instead, the lesion appeared soft, prompting consideration of soft tissue tumors in the differential diagnosis, including carcinoid tumors [5,7]. Intraoperative findings revealed multiple distinct, round, soft tissue masses. Histopathological examination confirmed the diagnosis of chondroma. The absence of a hard texture and the presence of multiple lesions distinguish this case from previously reported chondromas of the external auditory canal, suggesting the possibility of a unique clinical presentation or a different pathogenesis.

Extraskeletal chondroma is composed of mature hyaline cartilage that may be accompanied by calcification [1]. For this reason, CT imaging may demonstrate high attenuation values similar to those of bone. However, in the present case, no such findings were observed on CT, and no calcification was identified in the pathological specimen. The absence of calcification suggests that the tumor had an elastic, soft consistency.

Surgical excision is the recommended treatment for chondromas. Because malignant transformation is rare [8], careful monitoring may be appropriate in asymptomatic cases without evidence of hearing loss, tumor progression, otitis externa, or cerumen impaction. A surgical excision was performed for her complaint of left-sided hearing loss and for diagnosis. While recurrence of chondromas in the external auditory canal has not been reported, recurrence and malignant transformation have been observed in other anatomical sites [1,9]. Therefore, long-term follow-up is necessary to monitor for potential recurrence or pathological progression.

## Conclusions

Extraskeletal chondroma is exceedingly rare in the external auditory canal, and this represents the first documented case originating in the middle ear. The patient complained of conductive hearing loss on the left side. The tumor was removed surgically using transcanal endoscopic ear surgery under general anesthesia. Careful monitoring may be appropriate in asymptomatic cases because malignant transformation of extraskeletal chondroma is rare. While recurrence of chondromas in the external auditory canal has not been reported, recurrence and malignant transformation have been observed in other anatomical sites. Therefore, long-term follow-up is necessary to monitor for potential recurrence or pathological progression.
